# The Use of Cefiderocol in Gram-Negative Bacterial Infections at International Medical Center, Jeddah, Saudi Arabia

**DOI:** 10.3390/antibiotics13111043

**Published:** 2024-11-04

**Authors:** Reham Kaki, Amjad Taj, Sultan Bagaaifar

**Affiliations:** 1 Department of Medicine, King Abdulaziz University, Jeddah 21589, Saudi Arabia; 2 Department of Medicine, International Medical Center, Jeddah 21589, Saudi Arabia; aellahi@imc.med.sa (A.T.);; 3 Department of Infectious Disease, King Abdulaziz University, Jeddah 21589, Saudi Arabia; 4 Department of Infectious Disease, International Medical Center, Jeddah 21589, Saudi Arabia

**Keywords:** MDR Gram-negative bacteria, cefiderocol, antibiotics, comorbidity index

## Abstract

Background/Objectives: The necessity for ground-breaking treatments for Gram-negative infections is evident. The World Health Organization, the Infectious Diseases Society of America, and the European Commission have highlighted the critical insufficiency of efficient antibiotics, urging pharmaceutical businesses to manufacture new antibiotics. Therefore, developing new antibiotics with broad efficacy against Gram-negative pathogens is essential. Thus, this research aimed to evaluate the safety and effectiveness of cefiderocol in treating multidrug-resistant Gram-negative bacterial infections at the International Medical Center (IMC), Jeddah, Saudi Arabia. Methods: A retrospective analysis was conducted on patients treated from January 2021 to February 2023. Thirteen case groups treated with cefiderocol were compared to twenty control groups treated with other antibiotics. Results: The results indicated no statistically significant differences in ICU stay, comorbidity indices, or mortality rates between the two groups. Cefiderocol showed high clinical and microbiological cure rates, despite the severity of the patients’ conditions. Carbapenem-resistant *Klebsiella pneumoniae* and difficult-to-treat resistance *Pseudomonas aeruginosa* were the most prevalent pathogens in the case and control group, respectively. Two patients treated with cefiderocol developed *Clostridioides difficile* infection, emphasizing the need for close monitoring of potential adverse effects. Conclusions: The results of this study support cefiderocol as a viable alternative for managing serious infections instigated by multidrug-resistant Gram-negative bacteria.

## 1. Introduction

Cefiderocol (CFDC) is a unique siderophore cephalosporin approved for the treatment of Gram-negative bacterial infections (GNBI) [1]. It features a unique structure combining a cephalosporin core with a catechol siderophore side chain [2]. Siderophores are highly attractive, iron-chelating compounds that bacteria secrete to facilitate iron accumulation, which is crucial for their growth [3]. The structure of CFDC resembles both ceftazidime and cefepime, third- and fourth-generation cephalosporins, but CFDC possesses more stability against a wide range of beta-lactamases, including AmpC and Extended Spectrum Beta Lactamases (ESBL) [4]. CFDC uses the bacterial iron uptake system to enter cells, thus circumventing resistance mechanisms like porin loss or efflux pumps [1,5]. Once inside a cell, CFDC disrupts cell wall synthesis, leading to bacterial death [1,5]. It is effective against Ambler class A, C, and D beta-lactamases, including metallo-beta-lactamases (class B), and effective against multi-drug resistant (MDR) GNBI, including MDR *Enterobacterales*, *Pseudomonas aeruginosa*, *Acinetobacter baumannii*, and *Stenotrophomonas maltophilia* [1,5,6]. However, CFDC also has side effects such as gastrointestinal disorders, particularly diarrhea (4%) and constipation (3%). The observed side effects include hypertension (4%) and infusion site pain (3%), while vomiting, headache, hypokalemia, nausea, and cough are also observed, but affecting less than 2% [7]. Nevertheless, the side effect profile of CFDC is surpassed by its benefits, as in other cephalosporin antibiotics [8]. Consequently, the drug has demonstrated appropriate tolerability and safety [1,2]. CFDC is excreted renally, necessitating dosage adjustments based on renal function [4].

Gram-negative bacterial infections (GNBI) represent a significant global health threat, responsible for increasing numbers of intricate nosocomial pneumonia, urinary tract infections (UTIs), and bloodstream infections (sepsis) [1,2]. Extensively drug-resistant (XDR) and pan-drug-resistant Gram-negative bacteria are being documented with global increasing frequency [9,10]. The escalation of antibiotic resistance poses a worldwide crisis, complicating the selection of effective antibiotics for resistant pathogens [11]. Resistance mechanisms include beta-lactamases, carbapenemases, AmpC enzymes, efflux pumps, and porin mutations or loss [1,5]. While beta-lactamase inhibitor amalgamations (such as ceftazidime/avibactam, ceftolozane/tazobactam, meropenem/vaborbactam, and imipenem–cilastatin/relebactam) can counter some resistance mechanisms, they lack stability against metallo-beta-lactamases [1]. However, cefiderocol and aztreonam–avibactam are favorable alternatives for metallo-beta-lactamase producers [12]. The Centers for Disease Control and Prevention (CDC) and the World Health Organization (WHO) have categorized third-generation cephalosporin-resistant *Enterobacterales*, carbapenem-resistant *Enterobacterales* (CRE), *A. baumannii*, and *P. aeruginosa* with difficult-to-treat resistance (DTR-*P. aeruginosa*) as urgent threats needing new treatments [13,14].

The International Medical Center (IMC) in Jeddah, Saudi Arabia, is a multidisciplinary hospital with a 300-bed capacity, providing a wide range of comprehensive treatment options in a patient-centered care environment. The hospital emphasizes a holistic approach to medicine, addressing patients’ bodies, minds, and souls, with services delivered by over 150 physicians across the US, Canada, and Europe and more than thirty specialties. The state-of-the-art facilities at IMC make it one of the leading healthcare providers in the region [15].

In a Phase III study, CREDIBLE-CR, CFDC showed a higher cure rate for CRE Gram-negative pneumonia, urinary tract infections, and sepsis compared to the best possible treatments; however, all-cause mortality was more substantial in patients treated with CFDC [16]. CFDC has shown superior potency over ceftazidime/avibactam and meropenem against *A. baumannii*, such as MDR isolates. It exhibited greater effectiveness than ceftazidime–avibactam against meropenem-resistant and *Klebsiella pneumoniae* (*K. pneumoniae*) carbapenemase-producing *Enterobacterales*. CFDC demonstrated greater efficacy compared to ceftazidime–avibactam and meropenem against all resistance types of *P. aeruginosa* and *S. maltophilia* [4]. According to the APEKS-NP trial, CFDC was equally effective as high-dose, extended-infusion meropenem regarding all-cause mortality in patients with GNBI, with similar tolerability [17]. In randomized, double-blind clinical trials, CFDC was non-inferior to imipenem/cilastatin for treating intricate urinary tract infections and to meropenem for nosocomial pneumonia [1].

CFDC demonstrated strong efficacy against GNBI, which produces all four Ambler classes of beta-lactamases, including ESBL and carbapenemases. Susceptibility rates for ESBL-producing *Enterobacterales* ranged from 94–100% with CFDC [1]. CFDC maintained stability against the chromosomal AmpC beta-lactamases of *P aeruginosa* and *E. cloacae* (*Enterobacter cloacae*), with a low tendency to trigger AmpC production in these strains [1]. Additionally, CFDC demonstrated stronger in vitro antimicrobial activity against carbapenem-resistant strains of GNBI than other antibiotics [18]. The in vitro activity of CFDC correlates proficiently with its bactericidal effects in murine models of GNBI produced by *Enterobacterales*, *P. aeruginosa*, *A. baumannii*, and *S. maltophilia* [1,19].

Given the increasing incidence of GNBI in hospital settings and the difficulty in treating these infections with currently available antibiotics due to resistance, CFDC emerges as a promising option. At our center, CFDC is now available, and we aimed to measure its real-world efficacy in our patients. This research aimed to evaluate the effectiveness of CFDC in treating patients with complicated UTIs, pneumonia, central line infections, or abdominal infections caused by MDR, including CRE and DTR-*P. aeruginosa*, *A. baumannii*, and *S. maltophilia*, by observing clinical and microbiological cure rates, culture conversion if applicable, and mortality rates, comparing these results with those from similar patients treated with other antibiotic combinations before CFDC was available.

## 2. Results

A total of 13 and 20 patients from the case group and control group were incorporated into this research, respectively. Case group patients received cefiderocol during their stay in the hospital while the control group patients were treated with other antibiotics. The average age of the case group patients was 72.23 years with a standard deviation of 10.45 whereas the control group patients had a mean age of 72.40 years with a standard deviation of 9.86. The results indicated that the mean ages of the case group and control group had no statistically significant difference since the *p*-value (*p* = 0.96) exceeded the level of significance of 0.05. Therefore, both groups had a majority of males (69.2% in the case group and 55% in the control group). Thus, no statistically significant difference was found across the sex distribution of cases and control groups, *p*-value of 0.41. The severity of comorbidities can be observed through the Charlson index. The case group had moderate (38.5%) and severe (61.5%) scores of the Charlson index while 70% of the control group patients had severe scores and 10% and 20% had mild and moderate scores of the Charlson index. However, there was no statistically significant difference between the Charlson index in the case and control group patients (*p* = 0.306). In addition, a large variation was found in the length of stay of all patients. However, the length of stay did not have a statistically significant difference between cases and controls, with a *p*-value of 0.94. Similarly, the comparison between the case and control groups concerning ICU stay did not demonstrate any statistical difference (*p* = 0.84) (Table 1).

Table 2 shows the association between the case and control groups concerning indwelling catheters and mechanical ventilation. It is observed that the case group (92.3%) had a higher percentage of patients with indwelling catheters compared to the control group (75.0%), but the difference was not statistically significant (*p* = 0.208). Similarly, there was no statistical difference between the case and control groups regarding mechanical ventilation (*p* = 0.51). The case group (38.5%) had a lower percentage of patients on mechanical ventilation than the control group (50%).

Table 3 compares the clinical characteristics of the case and control groups. In the case group, 7.7% of patients had COVID-19: 61.5% had diabetes mellitus, renal impairment, and heart disease; 76.9% had hypertension; and only 15.4% each had chronic liver disease and solid organ malignancies (brain tumor and prostate cancer). In the control group, 10% of patients had COVID-19; 60% had diabetes mellitus, 50% had renal impairment, and 65% had heart disease; 80% had hypertension; and only 5% and 10% had solid organ malignancies (colon adenocarcinoma) and chronic liver disease, respectively. Moreover, 15.4% of patients in the case group developed *Clostridioides difficile* infection post-treatment with cefiderocol, while none in the control group did. It is observed that for each clinical characteristic examined (COVID-19, diabetes mellitus, hypertension, renal impairment, heart disease, chronic liver disease, and solid organ malignancies), the differences between the case group and control group were not statistically significant, as specified by the *p*-values (all above 0.05). This suggests there were no significant differences in these clinical features across the two groups.

**Figure 1:**
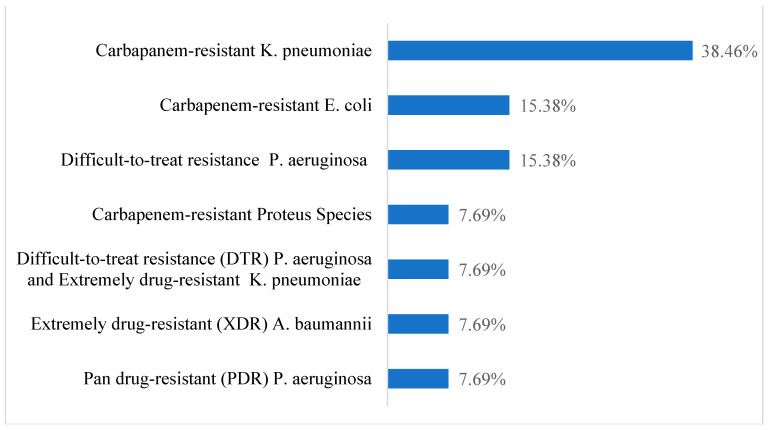
Microorganisms responsible in case group.

**Figure 2:**
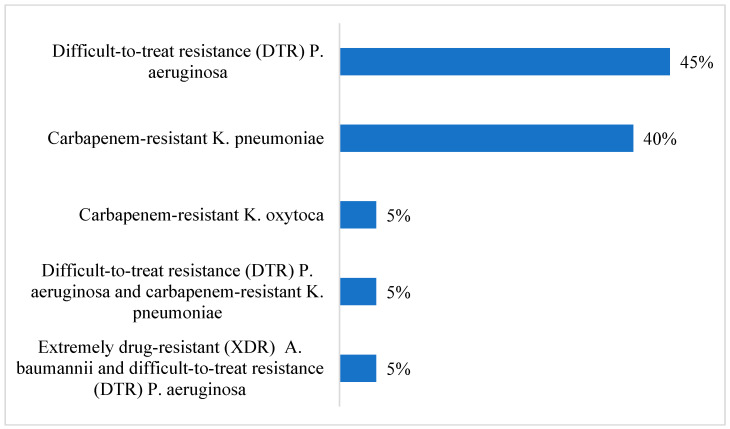
Microorganisms responsible in control group.

The commonest microorganism was carbapenem-resistant *K. pneumoniae*, found in 5 (38.5%) cases. The commonest organism in the control group was DTR *P. aeruginosa*, found in 45% of patients (Figure 1 and Figure 2).

Table 4 and Table 5 present a comparison between the case group and the control group concerning the type of infection, enzymes, phenotype, and the presence of resistance genes. Concerning infection types, pneumonia was noticed in 53.8% of the case group and 65.0% of the control group. Urinary tract infections (UTI) were more frequent in the case group (46.2%) compared to the control group (25.0%). Necrotizing fasciitis infections were not found in the case group but were presented in 10.0% of the control group. However, the results indicate insignificant differences between the two groups regarding infection type (*p*-value = 0.284). Considering the production of different enzymes, NDM was detected in 30.8% of the case group, significantly higher than the 5.0% in the control group (*p*-value 0.052). Both groups had a small percentage of individuals with New Delhi metallo-beta-lactamase (NDM) and *K. pneumoniae* carbapenemase (KPC) (7.7% in cases, 5.0% in controls). Oxacillinase (OXA-48) was not found in the case group but existed in 40.0% of the control group. The combination of OXA-48 and NDM was seen in 7.7% of the case group and not present in the control group. Verona integron-encoded metallo-beta-lactamase (VIM) was present in 7.7% of the case group but absent in the control group. XDR was not detected in the case group but was found in 5.0% of the control group. Whereas, regarding gene resistance, no resistance genes were detected in 46.2% of the case group and 45.0% of the control group, indicating no statistically significant difference was found (*p* = 0.052).

**Figure 3:**
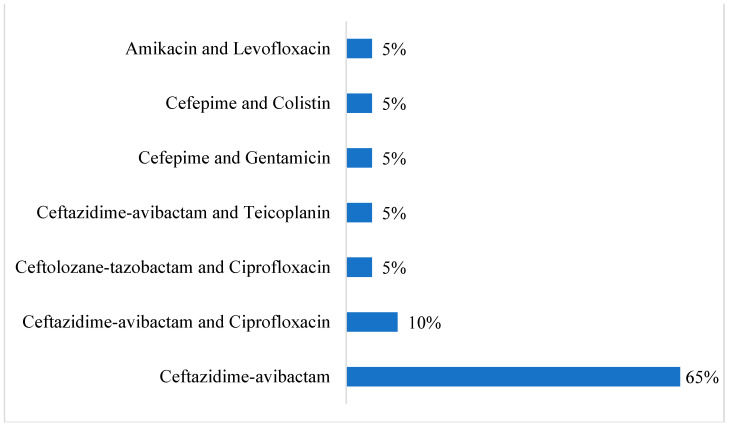
Antibiotic combination used in the control group.

Figure 3 illustrates the distribution of various antibiotic treatments used in the control group. It is observed that ceftazidime–avibactam was the most commonly used antibiotic treatment in the control group as it was administered to 65% of patients. Further, ceftazidime–avibactam and ciprofloxacin used in combination was the second most used treatment administered to control group patients (10% of patients).

Table 6 compares the 30-day mortality rates between the control group and the case group. In the case group, 76.9% (10 individuals) were alive after 30 days, while 15.4% (2 individuals) had died, and 7.7% (1 individual) had missing information regarding their mortality status. Similarly, in the control group, 75.0% (15 individuals) were alive, 15.0% (3 individuals) had died, and 10.0% (2 individuals) had missing information. The *p*-value of 0.975 indicates no significant difference in 30-day mortality rates between the two groups.

## 3. Discussion

This paper explored the safety and effectiveness of cefiderocol to treat MDR Gram-negative bacterial infections compared to other antibiotics. The necessity for innovative treatments for GNBI, particularly those involving metallo-beta-lactamase or OXA carbapenemase producers, is evident. The WHO, the Infectious Diseases Society of America, and the European Commission have highlighted the critical insufficiency of efficient antibiotics, urging pharmaceutical businesses to manufacture new antibiotics [13]. Therefore, developing new antibiotics with broad efficacy against Gram-negative pathogens, such as cefiderocol, is essential. Cefiderocol has revealed strong in vitro effectiveness against carbapenem-resistant *Enterobacterales*, carbapenem-resistant *A. baumannii*, *S. maltophilia, P. aeruginosa*, and even DTR-P [20,21,22].

To achieve the objective, this study included 13 patients in the case group treated with cefiderocol and 20 in the control group treated with other antibiotics. The demographic characteristics, including the age and sex distribution, were comparable among the groups. Further, there was no statistical difference between the clinical characteristics of the groups. Consequently, the microbial profiles showed that the most common organism in the case group was carbapenem-resistant *K. pneumoniae*, while in the control group, it was difficult-to-treat resistance (DTR) *P. aeruginosa*. The NDM gene was the most prevalent resistance gene in the cases, whereas the OXA-48 gene was most observed in controls. The study of Yassin et al. [23], highlights the global challenge of managing MDR Gram-negative infections. The study of Huang et al. [24] supported our study’s outcomes that *K. pneumoniae* is among the most common microorganism in the world and they found a carbapenem-resistant strain in 44.4% of isolates. In addition, studies from Saudi Arabia have reported similar trends in the production of different enzymes among Gram-negative bacteria, emphasizing the prevalence of NDM and OXA-48 genes in the region [25,26]. However, Zowawi et al. [26] reported the prevalence of OXA-48 type producers among carbapenem-resistant *Enterobacterales* in Saudi Arabia, which aligns with our control group’s microbial profile. On the other hand, our study found NDM was the most prevalent resistance gene in the case group. These findings supported by the results of Memish et al. [25] on carbapenemase production among Gram-negative bacteria in Saudi Arabia also found a high prevalence of NDM.

The effectiveness and safety profile of cefiderocol exhibited in our study are consistent with findings from international clinical trials. A Phase III study by Bassetti et al. [6] observed that cefiderocol shows a higher rate of cure for serious infections produced by carbapenem-resistant bacteria compared to the best possible treatment; nevertheless, the all-cause mortality rates were similar. Their results supported our findings that the survival rate in the cefiderocol group (control group) was higher than for other antibiotic treatments (case group). Corcione et al. [27] also found that a clinical cure was accomplished in 75% of cefiderocol-treated patients, while for combination therapy it was only 64.29%, supporting the use of cefiderocol as an effective treatment option.

A critical aspect of our study was the comparison of clinical outcomes between the cefiderocol-treated cases and the control group. All patients in the case group received infectious disease consultation, relative to only 80% in the control group. This comprehensive approach in the case group likely contributed to the observed outcomes, emphasizing the importance of specialized infectious disease management in treating MDR infections. Two case group patients developed *Clostridioides difficile* infection post-treatment with cefiderocol, while none in the control group did. Satlin et al. [28] also highlighted the adverse effects associated with cefiderocol, as they found one patient developed *Clostridioides difficile* infection, which resolved after intervention with oral vancomycin. This finding, although isolated, highlights the need for close monitoring of potential adverse effects when using novel antibiotics. The study of Miller et al. [29] has also supported our results, where patients treated with new antibiotics, including cefiderocol, were observed to have potential risks of *Clostridioides difficile* infection due to the broad-spectrum nature of these treatments. Further, this study highlighted the safety profile of cefiderocol and reported notable cases of *Clostridioides difficile* infections, emphasizing the importance of monitoring and managing such adverse effects during and after treatment. Deshpande et al. [30] also highlighted the association of broad-spectrum antibiotics, including clindamycin, fluoroquinolones, and cephalosporins, with an increased risk of *Clostridioides difficile* infection, supporting our findings.

Regarding antibiotic combinations used in the control group, ceftazidime–avibactam was the most common (65%), reflecting its widespread use in managing MDR Gram-negative infections. This aligns with international treatment patterns, where combinations of beta-lactam/beta-lactamase inhibitors are frequently employed as frontline therapies against resistant pathogens. However, the similar survival rates between the cefiderocol and control groups in our study suggest that cefiderocol can be a viable alternative, especially in cases where conventional treatments fail. This finding is supported by Wunderink et al. [17], where CFDC was equally effective as high-dose, extended-infusion meropenem regarding all-cause mortality at day 14 in patients with Gram-negative nosocomial pneumonia, with comparable tolerability. These findings advocate that cefiderocol is a promising alternative to treat patients with nosocomial pneumonia, including those affected by MDR Gram-negative bacteria.

Our study revealed a statistically insignificant difference in ICU stay and comorbidity indices between the cefiderocol-treated cases and the control group, indicating that cefiderocol’s efficacy is not influenced by the severity of the patient’s baseline condition. This finding aligns with the study of Sajib et al. [31], which also found that cefiderocol was effective in treating infections produced by carbapenem-resistant organisms in severely ill patients, with a high rate of clinical and microbiological cure. This emphasized the high efficiency of cefiderocol in managing difficult-to-treat infections. In our study, the most prevalent pathogens were carbapenem-resistant *K. pneumoniae* and DTR *P. aeruginosa*, similar to the study of Sajib et al. [31], which reported *P. aeruginosa* and *A. baumannii* as prevalent pathogens. The overall survival rates in our study were comparable to those reported by Sajib et al. [31], who found an all-cause mortality rate of 13.6% at 28 days. These findings collectively suggest that cefiderocol is a viable option for treating severe MDR infections, with a relatively safe profile and consistent efficacy across different patient populations.

The results of our study are contributing to expanding the body of evidence in support of cefiderocol’s role in treating MDR Gram-negative infections, particularly in regions with a high prevalence of carbapenem-resistant pathogens like Saudi Arabia. The consistent microbial profiles and resistance gene distributions between our study and those reported in the Saudi literature underscore the relevance of our findings to local clinical practice.

This study had several limitations and strengths. Among the strengths, the comprehensive evaluation of patient demographics, comorbidities, and clinical outcomes provided a robust dataset for analysis. Additionally, it was the first study in Saudi Arabia to evaluate cefiderocol in a real-world setting, contributing valuable data to the limited global literature on this novel antibiotic. However, this study also had limitations. The limited sample size may hinder the generalizability of the outcomes. The retrospective nature of the research might introduce biases related to data collection and accuracy. Differences in baseline characteristics, though not statistically significant, could still impact the outcomes and interpretations.

In the future, researchers should emphasize larger prospective cohort studies to validate these findings and offer more conclusive evidence on the effectiveness and safety of cefiderocol. Additionally, investigations into the cost-effectiveness and long-term impact of cefiderocol on antibiotic resistance patterns are warranted. The exploration of combination therapies involving cefiderocol and other antibiotics could also provide insights into optimizing treatment protocols for MDR infections.

## 4. Materials and Methods

### 4.1. Study Design

This retrospective study focused on IMC patients with resistant organisms from 1 January 2021 to 28 February 2023. We identified patients who used CFDC from the pharmacy records using the Healthcare Information System and Hakeem System in our hospital, then gathered data on resistant organisms from microbiology results, including sputum, urine, and blood cultures. We then compared the mortality rate with those of patients treated with other antibiotics for the same organisms.

### 4.2. Study Participants and Data Collection

This study incorporated thirteen patients in the case group treated with cefiderocol and twenty patients in the control group treated with other antibiotics. The data collection included patient demographics, comorbidities, site of infection, duration of hospital stay, type and use of antibiotic in the control group, and clinical outcomes in terms of mortality within 30 days.

### 4.3. Inclusion and Exclusion Criteria

- Inclusion criteria: males and females, ages 18–80, hospitalized as inpatients or in the ICU, and receiving CFDC for MDR organisms.
- Exclusion criteria: patients receiving antibiotics with similar microbial coverage as CFDC for GNBI to minimize interference with the results.

### 4.4. Collection of Specimens and Culture Media

#### 4.4.1. Culture Media

This study utilized several types of culture media obtained from Saudi Prepared Media Laboratory Company Ltd (Riyadh, Saudi Arabia). MacConkey agar with crystal violet was used as a differential and selective medium to enhance the growth of *Enterobacterales* members and to differentiate lactose fermenters from non-fermenters. Chocolate agar and sheep blood agar, both enriched media, were employed to support the rapid and robust growth of a wide range of pathogens.

#### 4.4.2. Sample Inoculation Procedure

Samples were inoculated under aseptic conditions inside a Class A2 safety cabinet. Sterile loops (0.001 mL) were used to streak the samples onto sheep blood agar, chocolate agar, and MacConkey agar. The inoculated plates were then incubated aerobically at 35 °C for 48 h.

### 4.5. Identification of Isolated Bacteria

#### 4.5.1. Gram Stain Procedure

The Gram stain procedure was performed according to standard methods as outlined by Cheesbrough et al. [32].

#### 4.5.2. Vitek-2 System

The Vitek-2 automated susceptibility system, manufactured by bioMérieux in the USA, was employed for bacterial identification and antimicrobial susceptibility testing. This system is designed for clinical microbiology laboratories, offering high levels of automation and capacity, along with automatic pipetting and dilution capabilities. Microorganism suspensions are introduced onto identification cards through a built-in vacuum system. The suspension is held in a test tube that is positioned in a designated rack, with the identification card placed next to it, and a transfer tube is inserted into the suspension tube. The filled cassette, which can accommodate up to 15 tests, is automatically transported into the station of the vacuum chamber. The suspension containing the organism is pushed through the transfer tube into micro-channels to fill all of the test wells as the vacuum is activated and the air is allowed back in.

### 4.6. Materials

The materials used included test tubes, Vitek racks, sterile loops, 0.45% saline, and a turbidity meter.

#### 4.6.1. Procedure for Vitek-2

The Vitek-2 system was used for bacterial identification and antimicrobial susceptibility testing according to standard procedures [33].

#### 4.6.2. Weekly Vitek-2 Quality Control

For quality control (QC), these American Type Culture Collection (ATCC) strains were used for identification: *E. hormaechei* ATCC 700323, *S. maltophilia* ATCC 17666, *E. casseliflavus* ATCC 700327, and *S. saprophyticus* ATCC BAA750. For sensitivity QC, the following ATCC strains were used: *P. aeruginosa* ATCC 27853, *E. coli* ATCC 25922 and ATCC 35218, *K. pneumoniae* subsp. *pneumoniae* ATCC 700603 (National Committee for Clinical Laboratory Standards selected *K. pneumoniae* ATCC 700603 as an ESBL quality control (QC) strain for approval testing that clinical laboratories can apply to enhance the detection of ESBLs in *K. pneumonia*), *S. aureus* ATCC 29213, *S. aureus* (Cefoxitin screen) ATCC BAA1026, *S. aureus* (ICR Test) ATCC BAA977 and ATCC BAA976, *E. faecalis* ATCC 29212, and *E. faecalis* ATCC 51299.

#### 4.6.3. Media Quality Control

Blood agar QC was performed on two plates upon receiving a new shipment; one tested for sterility and the other for media quality using *S. aureus* ATCC 25923 and *E. coli* ATCC 25922.

For MacConkey agar QC, two plates from each new shipment were tested: one for sterility and the other for media quality using *P. aeruginosa* ATCC 27853 and *E. coli* ATCC 25922.

For chocolate agar QC, two plates from each new shipment were tested: one for sterility and the other for media quality using *H. influenzae* ATCC 9007 and *N. gonorrhoeae* ATCC 19424.

### 4.7. Data Analysis

To perform the data analysis, we employed SPSS version 27. The mean and standard deviation were estimated for quantitative variables, while frequency and percentages were computed for qualitative variables. The relationship between the categorical variables was examined by employing the Chi-square test; a *p*-value less than 0.05 was considered significant. The association between quantitative variables was estimated by applying an independent t-test. Data are presented in charts and tables.

## 5. Conclusions

This study demonstrated that cefiderocol is a promising option for treating MDR Gram-negative infections, with outcomes comparable to other antibiotic regimens. The similar survival rates and absence of significant differences in other clinical parameters suggest that cefiderocol can be a viable alternative in settings with high resistance rates. Our results contribute toward the expansion of the literature in support of the use of cefiderocol as a critical instrument in the fight against antibiotic resistance.

## Figures and Tables

**Table 1:** Comparison between case and control groups regarding demographic characteristics.

Demographic Characteristics	Category	Case Group (n = 13)		Control Group (n = 20)		*p*-Value
		Frequency	%	Frequency	%	
Gender	Male	9	69.2	11	55.0	0.414 *
	Female	4	30.8	9	45.0	
ICU Stay	Yes	5	38.5	7	35.0	0.840 *
	No	8	61.5	13	65.0	
Charlson Index	Mild	0	0	2	10.0	0.306 *
	Moderate	5	38.5	4	20.0	
	Severe	8	61.5	14	70.0	
Age	Mean ± SD	72.23 ± 10.450		72.40 ± 9.859		0.963 ^+^
Length of Hospital Stay	Mean ± SD	40.54 ± 55.667		42.25 ± 63.649		0.937 ^+^

Note: Intensive care unit (ICU). *: Chi-square test, ^+^: Independent *t*-test.

**Table 2:** Comparison between case and control groups regarding indwelling catheters and mechanical ventilation.

	Category	Case Group (n = 13)		Control Group (n = 20)		*p*-Value
		Frequency	%	Frequency	%	
Indwelling Catheter	Yes	12	92.3	15	75.0	0.208 *
	No	1	7.7	5	25.0	
Mechanical Ventilation	Yes	5	38.5	10	50.0	0.515 *
	No	8	61.5	10	50.0	

*: Chi-square test.

**Table 3:** Comparison between Case and Control Group regarding Clinical Characteristics.

Clinical Characteristics	Category	Case Group (n = 13)		Control Group (n = 20)		*p*-Value
		Frequency	%	Frequency	%	
COVID-19	Yes	1	7.7	2	10.0	0.975 *
	No	10	76.9	15	75.0	
	Not-tested	2	15.4	3	15.0	
Diabetes miletus	Yes	8	61.5	12	60.0	0.930 *
	No	5	38.5	8	40.0	
Hypertension	Yes	10	76.9	16	80.0	0.833 *
	No	3	23.1	4	20.0	
Renal impairment	Yes	8	61.5	10	50.0	0.515 *
	No	5	38.5	10	50.0	
Heart disease	Yes	8	61.5	13	65.0	0.840 *
	No	5	38.5	7	35.0	
Chronic liver disease	Yes	2	15.4	2	10.0	0.643 *
	No	11	84.6	18	90.0	
Solid organ malignancies	Brain Tumor	1	7.7	0	0	0.282 *
	Colon Adenocarcinoma	0		1	5.0	
	Prostate Cancer	1	7.7	0	0	
	None	11	84.6	19	95.0	
*Clostridioides difficile* infection	Yes	2	15.4	0	0	0.070 *
	No	11	84.6	20	100	

Note: Coronavirus (COVID-19). *: Chi-square test.

**Table 4:** Comparison between case and control group regarding type of infection.

Category		Case Group (n = 13)		Control Group (n = 20)		*p*-Value
		Frequency	%	Frequency	%	
Type of Infection	Pneumonia	7	53.8	13	65.0	0.284 *
	UTI	6	46.2	5	25.0	
	Necrotizing fasciitis	0	0	2	10.0	

Note: Urinary tract infection (UTI). *: Chi-square test.

**Table 5:** Comparison between case and control group regarding types of enzymes produced, phenotype, and resistance genes.

Category		Case Group (n = 13)		Control Group (n = 20)		*p*-Value
		Frequency	%	Frequency	%	
Enzymes	NDM	4	30.8	1	5.0	0.052 *
	NDM and KPC	1	7.7	1	5.0	
	OXA-48	0	0	8	40.0	
	OXA-48 and NDM	1	7.7	0	0	
	VIM	1	7.7	0	0	
Phenotype	XDR	0	0	1	5.0	
Resistance Gene	No genes detected	6	46.2	9	45.0	

Note: New Delhi metallo-beta-lactamase (NDM), *K. pneumoniae* carbapenemase (KPC), Oxacillinase (OXA-48), Extensively drug-resistant (XDR), and Verona integron-encoded metallo-beta-lactamase (VIM). *: Chi-square test.

**Table 6:** Comparison between case and control group regarding mortality within 30 days.

Mortality Within 30-Days	Case Group (n = 13)		Control Group (n = 20)		*p*-Value
	Frequency	%	Frequency	%	
Alive	10	76.9	15	75.0	0.975 *
Dead	2	15.4	3	15.0	
Missing information	1	7.7	2	10.0	

*: Chi-square test.

## Data Availability

Data will be made available upon reasonable request from the corresponding author.
